# Pancreatic (pro)enzymes treatment suppresses BXPC-3 pancreatic Cancer Stem Cell subpopulation and impairs tumour engrafting

**DOI:** 10.1038/s41598-019-47837-7

**Published:** 2019-08-06

**Authors:** Pablo Hernández-Camarero, Elena López-Ruiz, Carmen Griñán-Lisón, María Ángel García, Carlos Chocarro-Wrona, Juan Antonio Marchal, Julian Kenyon, Macarena Perán

**Affiliations:** 1 Department of Health Sciences, University of Jaén, Jaén, Spain; 2 Biopathology and Regenerative Medicine, Institute (IBIMER), Centre for Biomedical Research (CIBM), University of Granada, Granada, Spain; 3 Biosanitary Research Institute of Granada (ibs. GRANADA), University Hospitals of Granada-University of Granada, Granada, Spain; 4 Department of Human Anatomy and Embryology, Faculty of Medicine, University of Granada, Granada, Spain; 5 Department of Biochemistry and Molecular Biology 3 and Immunology, University of Granada, Granada, Spain; 6 Excellence Research Unit “Modeling Nature” (MNat), University of Granada, Granada, E-18016 Spain; 7 The Dove Clinic for Integrated Medicine, Twyford, SO21 1RG UK

**Keywords:** Cancer stem cells, Drug development

## Abstract

Cancer stem cells (CSCs) subpopulation within the tumour is responsible for metastasis and cancer relapse. Here we investigate *in vitro* and *in vivo* the effects of a pancreatic (pro)enzyme mixture composed of Chymotrypsinogen and Trypsinogen (PRP) on CSCs derived from a human pancreatic cell line, BxPC3. Exposure of pancreatic CSCs spheres to PRP resulted in a significant decrease of ALDEFLUOR and specific pancreatic CSC markers (CD 326, CD 44 and CxCR4) signal tested by flow cytometry, further CSCs markers expression was also analyzed by western and immunofluorescence assays. PRP also inhibits primary and secondary sphere formation. Three RT^2^ Profiler PCR Arrays were used to study gene expression regulation after PRP treatment and resulted in, (i) epithelial-mesenchymal transition (EMT) inhibition; (ii) CSCs related genes suppression; (iii) enhanced expression of tumour suppressor genes; (iv) downregulation of migration and metastasis genes and (v) regulation of MAP Kinase Signalling Pathway. Finally, *in vivo* anti-tumor xenograft studies demonstrated high anti-tumour efficacy of PRP against tumours induced by BxPC3 human pancreatic CSCs. PRP impaired engrafting of pancreatic CSC’s tumours in nude mice and displayed an antigrowth effect toward initiated xenografts. We concluded that (pro)enzymes treatment is a valuable strategy to suppress the CSC population in solid pancreatic tumours.

## Introduction

Improving therapies against hematopoietic malignancies and solid tumours are making cancer a chronic disease and represent a short-term success. Nevertheless, among cancers, pancreatic ductal adenocarcinoma still has a very poor prognosis with short life expectancy. To decrease cancer mortality rate, strategies to prevent cancer recurrence are needed.

The discovery of a subpopulation of cells with stem cell-like features, called cancer stem cells (CSCs), in a wide spectrum of solid tumours^1^ has led to a new model of tumorigenesis, the CSCs hypothesis. This model suggests that the intrinsic characteristics of these cells, such as self-renewal and the ability to differentiate, drive tumour initiation and progression, and the subsequent chance of developing metastasis and tumour recurrence^2^. The CSCs hypothesis is based on the following findings: (i) CSCs represent a small fraction of cancer cells within a tumour; CSCs have tumorigenic potential when transplanted into immunodeficient mice; (ii) CSCs can be isolated by specific surface markers; (iii) tumours resulting from the CSCs contain mixed tumorigenic and non-tumorigenic cells of the original tumour; and (iv) CSCs can be serially transplanted through multiple generations, indicating that it is a self-renewing population^3,4^. An additional and very important feature of the CSCs population is their ability to remain quiescent for extended periods of time, and therefore evade conventional therapies (chemo- and radiotherapy) that are targeted to highly proliferative cells^5^. Consequently, a priority for improving cancer treatment and reducing the risk of cancer relapse is to develop new strategies that selectively target CSC eradication while sparing normal stem cells.

One of the biggest challenges in CSCs research has been the development of methods for culture, expansion, and analyses of undifferentiated cancer cells *in vitro*. In this respect, our group has experience in CSCs enrichment from cancer cell lines or from cancer cells isolated from patients. We have recently patented a method to isolate and culture CSCs and we have also implemented different studies to test drugs directed against CSCs^6^.

Previous research has suggested a putative utility of pancreatic (pro)enzymes in cancer treatment^7,8^. In agreement with these studies, we have shown *in vivo* and *in vitro* the anti-tumour efficacy of a novel pro-enzyme formulation consisting of a combination of Trypsinogen and Chymotrypsinogen A (PRP), that proves to be an effective and non-toxic treatment, able to induce lineage specific cellular differentiation, inhibit angiogenesis, tumour growth, cancer cell migration and invasiveness. Furthermore, a suppository formulation containing both pancreatic pro-enzymes increased the life expectancy of advanced cancer patients^9,10^. Based on these findings, we postulated that pancreatic pro-enzymes may induce differentiation of CSCs and reduce recurrence and metastatic spread. Here we have tested, *in vitro* and *in vivo*, the effect of the formulation PRP on CSCs isolated from the human pancreatic cancer cell line BxPC3. To do so, we first determined the effect of the anti-cancer drug on cell viability, proliferation and sphere formation capacity of CSCs isolated from BxPC3. Second, we analysed changes in pancreatic CSCs markers after treatment. Third, we have analysed with RT^2^ Profiler PCR Arrays changes of expression of genes involved in EMT or related to CSCs and MAP Kinase Signalling Pathway induced by PRP treatment. Four, we have studied expression of mirRNAs related to CSCs and to characterize the mechanism of action of the pro-enzyme formulation we have studied protein expression. Finally, we have conducted a xenograft study to assess the effect of PRP in suppressing tumour growth *in vivo*.

We can conclude that PRP reduces the CSC population, tumour initiation and decreases fibrotic tissue in tumours.

## Results

### PRP treatment reduces CSCs population in pancreatic cancer

**Figure 1:**
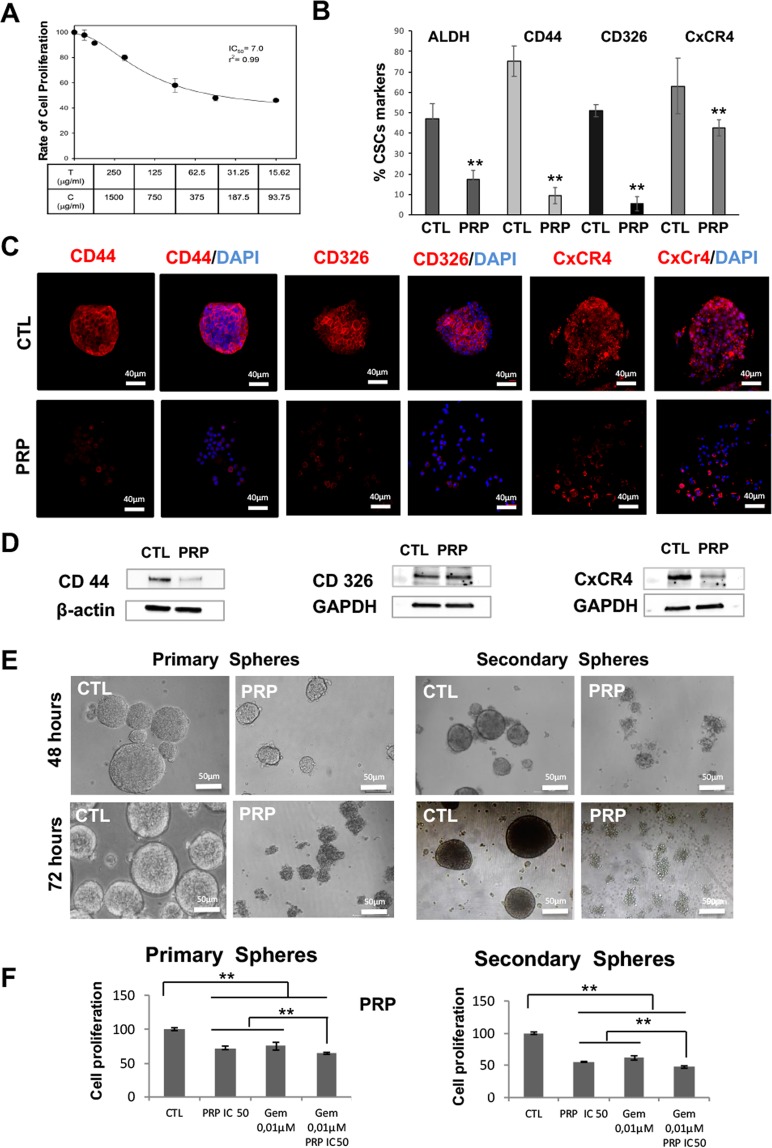
*In vitro* analysis of tumorigenic capacity of BXPC3 CSCs after PRP treatment. (**A**) Antiproliferative activity of PRP against CSCs from pancreatic BXPC3 cell line. (**B**) Decreased ALDH1 activity and CSCs markers expression: CD44, CD326 and CXCR4 in BXPC3 CSCs. (**C**) Representative confocal images of CD44, CxCR-4 and CD326 expression in BxPC3 treated and control CSCs. CD44, CD326 and CxCR-4 expression were detected in red and nuclei were counterstained with DAPI (blue). Scale bar represents 40 μm. (**D**) Western blot analysis of CD44, CD326 and CxCR-4 in BXPC3 CSCs treated with PRP versus non-treated. Β-actin and GAPDH were used as a internal controls. (**E**) Representative images of BXPC-3 primary and secondary spheres treated with PRP (T/C 0.07/0.42 mg/mL) at 48 and 72 hours after treatment. Scale bar represents 50 μm. (**F**) *In vitro* proliferation assay on BXPC-3 CSCs after treatment with PRP and gemcitabine. BXPC-3 primary and secondary spheres were incubated with PRP, gemcitabine (0,01 μM treatment) or with a combination of PRP/gemcitabine 72 h. The PRP, gemcitabine and combination of PRP and gemcitabine treatment resulted in a statistically significant decrease in primary and secondary CSCs spheres compared to control. Statistical significance indicated **p < 0.01. vs. samples not treated.

We have previously described the anti-tumor effect of PRP in some pancreatic, colon, oesophagus and ovarian cancer cell lines^9,10^. To test the selective action of PRP against pancreatic CSCs enriched subpopulations we first determined the IC_50_ by MTT assay (Fig. 1A). ALDH has been described as a marker for the identification of CSCs^11^. Here we detected ALDH positive cells by ALDEFLUOR assay using flow cytometry (Fig. 1B) and proved that treatment with PRP significantly reduces the population of ALDH positive cells (from 47.2% to 17.5%) in pancreatic CSCs. Furthermore, Fig. 1B shows that the percentage of cells that express specific pancreatic CSCs markers CD 44, CD 326 and CxCR4 significantly decreased after PRP treatment from 75.1% to 9.5%; from 51.2% to 5.5% and from 63.1% to 42.4% respectively, which signifies that PRP treatment reduces CSCs population in pancreatic cancer cells. Finally, we further determine the expression of the pancreatic cancer markers CD44, CD326 and CxCR4 by immunofluorescence (Fig. 1C) and by western blotting (Fig. 1D and Supplementary Fig. S1). Both assays demonstrate that PRP reduced the expression of specific pancreatic CSCs markers by a decrease in fluorescence red signal shown by treated cells when compared with control cells, which was corroborated by western blotting analysis.

### PRP inhibits spheroid formation by CSCs

We next tested if PRP could suppress sphere formation by pancreatic CSCs. To do so, PRP, was added to the medium at day 0, on cancer cells cultured under sphere conditions (conditioned medium and low attached culture wells) and compared with the effect of gemcitabine, one of the main chemotherapy drugs used to treat pancreatic cancer. Figure 1E shows images of cell cultures on day 2 and day 3. On day 2 it is clear that the addition of PRP to the medium significantly reduces the number and size of CSC spheres. In addition, the morphology of the spheres formed is markedly different, being regular in the control non-treated cells while it was quite irregular in PRP treated cells. On day 3, PRP treated cells do not form spheres and showed the appearance of non-healthy disaggregated cells. BXPC-3 primary spheres treated with PRP demonstrated inhibition of proliferation significantly different from the control group and similar to gemcitabine treatment. The combination treatment of PRP and gemcitabine resulted in a statistically significant decrease in primary CSCs compared to both PRP treatment alone (p < 0.01) and gemcitabine treatment alone (p < 0.01) (Fig. 1F). Moreover, PRP abolished secondary sphere-formation (Fig. 1E) an effect comparable to treatment with gemcitabine. There were statistically significant differences between formed secondary spheres when cells were treated with PRP or gemcitabine alone compared with the control group and between the PRP/gemcitabine combined treatment and all other experimental groups (p < 0.01). The highest rate of secondary sphere inhibition was observed in the group treated with PRP and gemcitabine (p < 0.01) (Fig. 1F).

### PRP treatment induces genome-wide gene expression changes in pancreatic CSCs

**Figure 2:**
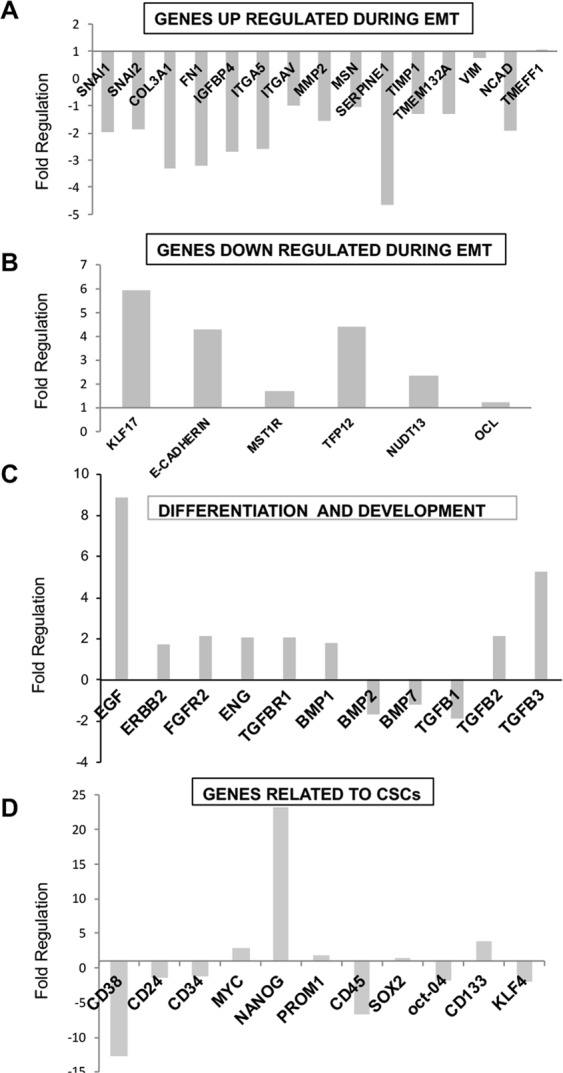
Gene expression profiling of BxPC3 CSCs treated with PRP using RNA Human EMT and CSCs RT2 Profiler PCR Array. BxPC3 CSCs were treated with PRP on day 2 and on day 4. On day 5, total RNA from treated and non-treated CSCs was extracted. A pool of three total RNAs extracted from three independent experiments was used for first strand cDNA synthesis. Gene expression was determined using RT^2^ Profiler PCR Array of EMT and CSCs. (**A**) Expression of genes up regulated during EMT. (**B**) Expression of genes down regulated during EMT. (**C**) Expression of genes related to differentiation and development. (**D**) Expression of genes related to CSCs. All gene expressions were normalised to the untreated control.

To understand the molecular changes underlying PRP exposure to pancreatic CSCs, we performed three PCR microarray analysis to measure regulation of the EMT process, CSCs characteristics and variations in Kinase Signalling Pathway related to the treatment. Figure 2A shows up regulation of selective genes that are down regulated during the EMT process. It is important to note that E-cadherin displayed a fold regulation of 4.3, indicating that PRP treatment clearly induces its expression. Furthermore, Kruppel-like-factor 17 (KLF17) a negative regulator of metastasis and EMT^12^ was also up-regulated by PRP treatment, reaching a fold regulation of 5.9. Other genes such as MST1R (1.6 folds); TFPI2 (4.4 folds) and NUDT13 (2.3 folds), significantly increased their expression after pro-enzyme treatment, implying potent anti-EMT effects of the PRP formulation. Genes that are up regulated during EMT were analysed and the results are summarized in Fig. 2B. Remarkably, 13 genes that have been described to induce EMT were down regulated with PRP treatment. Of interest, the transcription factor Snail which is up-regulated in metastatic cells that are undergoing EMT, was down regulated (SNAI1: −1.9 folds and SNAI2: −1.86 folds). In addition, the serine protease inhibitor SERPINE1, a poor prognostic biomarker in various cancers that promotes tumour progression^13^, presented a noticeable fold down regulation of −4.6. Furthermore, gene expression of the integrins ITGA5 (−2.5 folds) and ITGAV (−1.02 folds) were also down regulated.

In previous studies, we proved that PRP induces cell differentiation in the pancreatic cancer cell line Panc1^9^. Here, 7 genes related to cell differentiation were up-regulated after PRP treatment (Fig. 2C). Also, the expression of epidermal growth factor (EGF), related to beta cell differentation^14^, increased 8.9 folds when compared with control cells. Furthermore, pro-enzyme treatment of pancreatic CSCs induced down-regulation of 6 genes recognized as CSCs markers (Fig. 1D). Although we found a high up-regulation of Nanog (23 folds), interestingly other genes directly involved in the maintenance or acquisition of stemness such as Oct4 and KLF4 were down-regulated due to PRP addition. In addition, the specific CSCs markers CD38, CD45, CD24 and CD34 were down regulated (−12.8 folds; −6.7 folds; −1.4 folds and −1.2 folds respectively); while CD133 was up-regulated (3.9 folds) (Fig. 2D).

**Figure 3:**
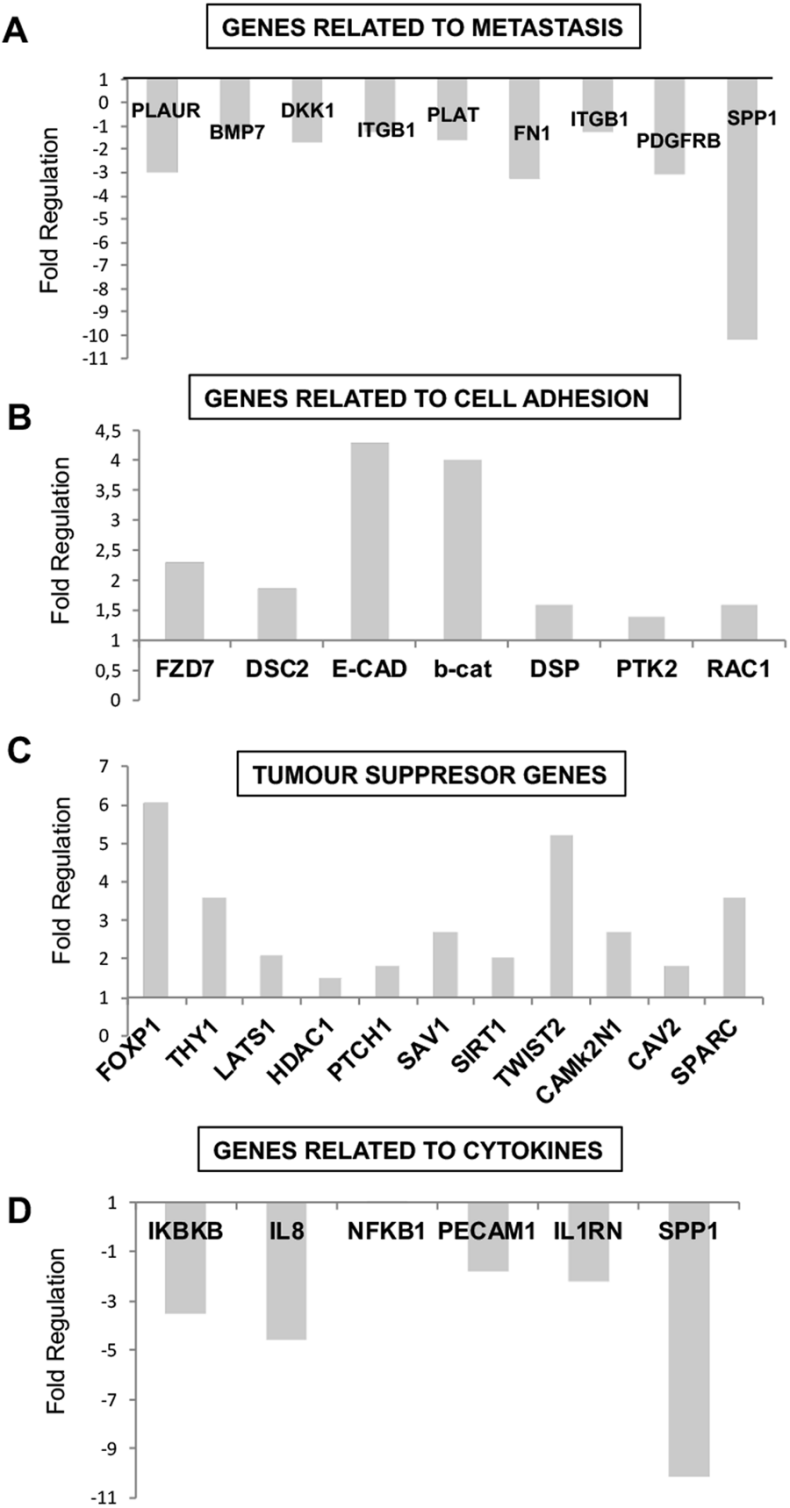
Human EMT and CSCs RT2 Profiler PCR Array of BxPC3 CSCs treated with PRP. BxPC3 CSCs were treated with PRP on day 2 and on day 4. On day 5, total RNA from treated and non-treated CSCs was extracted. A pool of three total RNAs extracted from three independent experiments was used for first strand cDNA synthesis. Gene expression was determined using RT^2^ Profiler PCR Array of EMT and CSCs. (**A**) Expression of genes genes related to metastasis. (**B**) Expression of genes related to cell adhesion. (**C**) Expression of tumour suppresor genes. (**D**) Expression of genes related to cytokines. All gene expressions were normalised to the untreated control.

Figure 3A shows that 8 genes implicated in metastasis and cell invasion were down-regulated by PRP treatment (PLAUR; BMP7; DKK1; ITGB1; PLAT; FN1; PDGFRB and SPP1) Furthermore, the expression of seven genes related to cell adhesion were up-regulated (Fig. 3B). It is important to note that the expression of E-cadherin (4.3 folds) and β-catenin (4 folds) was enhanced by pro-enzyme treatment in agreement with our previous studies^9^. In addition, 11 genes with a tumour suppressor role were up-regulated by pro-enzyme treatment, results are summarized in Fig. 3C. Remarkably there was enhanced expression of the transcription factor FOXP1 (6.04 folds), TWIST2 (5.2 folds) and THY1 (3.622 folds). In addition, SPARC, an extracellular protein involved in the deposition and modelling of the extracellular matrix that is downregulated in many tumour types was found to be up-regulated (3.6 folds) by PRP treatment. Furthermore, SIRT1, a member of the sirtuin family of proteins proven to inhibit proliferation of pancreatic cancer cells^15^, increased the expression in 2 folds in treated cells.

The expression of genes encoding cytokines was investigated after PRP treatment and compared with untreated pancreatic CSCs. Figure 3D shows down-regulation of IKBKB; IL8; PECAM1; IL1RN and SPP1. Remarkably, IL-8 (−4.5 folds) and Secreted phosphoprotein-1 (SPP1, −10 folds).

**Figure 4:**
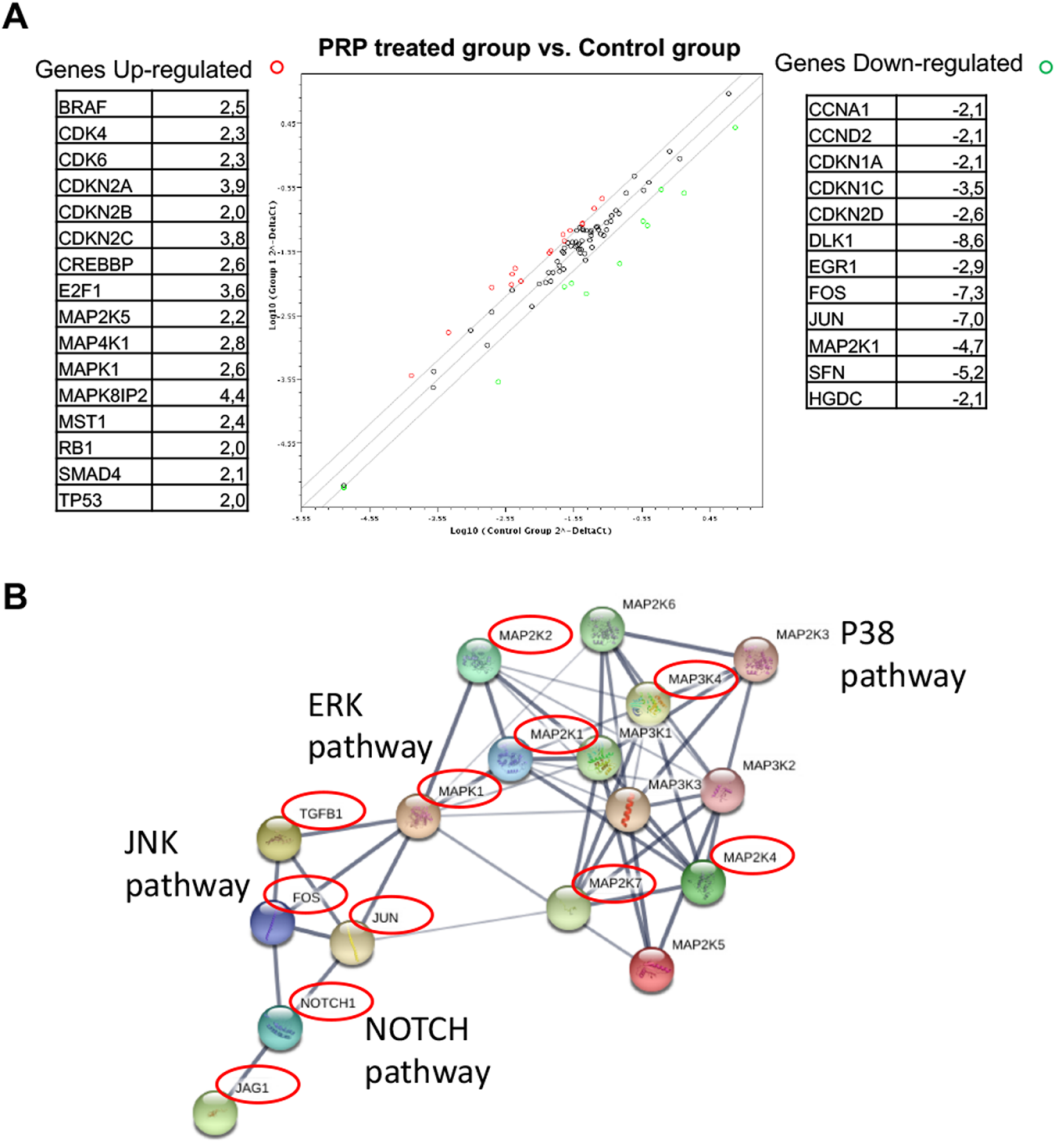
Screening of MAP Kinase Signalling Pathway genes by RT^2^ Profiler PCR Array. (**A**) BxPC3 CSCs were treated with PRP on day 2 and on day 4. On day 5, total RNA from treated and non-treated CSCs were extracted. A pool of three total RNAs extracted from three independent experiments was used for first strand cDNA synthesis. Gene expression was determined using MAP Kinase Signalling Pathway RT^2^ Profiler PCR Array. Sixteen genes were up-regulated, whereas twelve were downregulated compared to the controls. (**B**) Functional in-silico analysis of proteins related to the c-Jun N-terminal kinase (JNK) pathway. Grey lines represent protein-protein associations, line thickness indicates the strength of data support (active interaction sources from experimental and database only). Red circles highlights genes coding for those proteins that were downregulated by PRP treatment.

Finally, the Human MAP Kinase Signaling Pathway RT^2^ Profiler PCR Array was used to profile the expression of 84 genes related to the MAP kinase (MAPK) signalling pathway. As can be observed in Fig. 4A,B, genes related to JNK and ERK pathways are down-regulated as a consequence of PRP treatment, for instance relevant oncogenic transcription factors JUN (−7 folds), FOS (−7.3 folds) and MEK1 (MAP2K1) (−4.7 folds). Furthermore, PRP induced the up-regulation of cyclin-dependent kinase inhibitor genes (CDKN2A, 3.9 folds; CDKN2B, 2 folds and CDKN2C, 3.8 folds) and decreased the expression of DLK1 (−8.5 folds). In addition, SFN-1 (YWHAZ) gene was down regulated by PRP treatment (−5.2 folds). Moreover, we found p16; SMAD4 and TP53 to be up-regulated Fig. 4A.

Figure 4B summarizes in an in-silico analysis the relationship between some of the genes that were downregulated after PRP treatment, which are involved in the TGFβ SMAD-independent pathway, such as mitogen-activated protein kinases (MAPKs) including c-Jun N-terminal kinase (JNK), p38, and extracellular signal-regulated kinase (ERK) (reviewed by Solomon, 2014)^16^.

The expression levels of other genes in the microPCR panels did not significantly change after PRP treatment as is shown in Supplementary Fig S2.

### Q-PCR analyssis of Rac1, Rac1b and Smad7 gene expression in pancreatic CSCs treated with PRP

**Figure 5:**
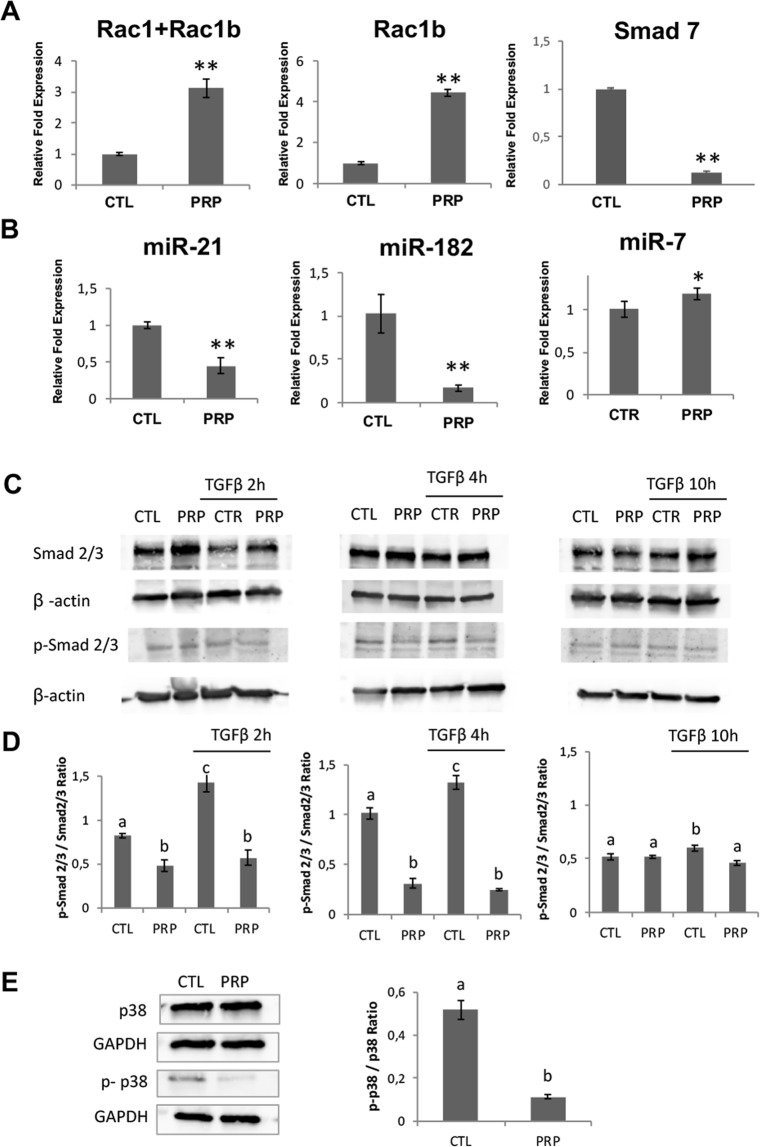
PRP treatment downregulated both Smad-dependent and Smad-independent pathways in TGFβ signaling. (**A**) RT-qPCR analysis of Rac1b; Rac1 + Rac1b and Smad 7 after PRP treatment. All gene expressions were normalised to the untreated control. (**B**) RT-qPCR analysis show the fold change of miR-21-5p, miR-182-3p and miRNA-7. Values were normalized using the UniSp6 RNA Spike-in control primer set. (**C**) Treated and not treated BxPC3 CSCs were incubated with TGFβ1 (5 ng/mL) for 2 h, 4 h and 10 h. The phosphorylation levels of Smad2/3 and total Smad2/3 were analyzed by western blotting at indicated time points. (**D**) The relative expression of p-Smad2/3 and total Smad2/3 normalized to β-Actin was quantified using Image-J software NIH and is shown as a ratio of p-Smad2/3 versus Smad2/3. (**E**) Western blotting analysis of p-p38 and total p38 expression in CSCs treated with PRP versus non-treated. The relative expression of p-p38 and total p38 normalized to GAPDH was quantified using Image-J software NIH and is shown as a ratio p-p38/p38 Different letters stand for significant differences (p < 0.05).

Since Rac1 is a key mediator of EMT in pancreatic cancer, and it has been shown that the Rac1-related isoform, Rac1b, acts as an endogenous inhibitor of Rac1 in TGFβ signalling, we analyzed Rac1b and Rac1 + Rac1b expression in PRP treated cells by RTqPCR. As demonstrated in Fig. 5A, Rac1 + Rac1b and Rac1b gene expression were significantly up-regulated following PRP treatment (2.1-fold and 3.4-fold respectively, p < 0.01) indicating that (pro)enzymes treatment may favours decreased expression of TGFβ1-induced tumorigenic events, including EMT, invasion, and metastasis.

In addition the expression of Smad7 (Fig. 5A) was significantly downregulated after PRP treatment (approximately 1 fold).

### PRP treatment change miRNA expression

To further explore the effect of PRP formulation on pancreatic CSCs, we analysed known pancreatic cancer miRNAs. Interestingly, we observed that miR-21-5p and miR-182-3p levels were significantly down-regulated after pancreatic CSCs were treated with PRP (Fig. 5B) (p < 0.01). In addition, results showed that treated CSCs increased expression of miRNA-7 (Fig. 5B) (p < 0.05).

### PRP treatment downregulates both Smad-dependent and Smad-independent pathways in TGFβ signaling

To support the claim that PRP regulates TGFβ pathway the expression of phosphorylated Smad2/3 was detected under TGFβ stimulation with time intervals in control and treated cells (Fig. 5C, Supplementary Figs S3 and 4 and Fig. 5D). Stimulation of control cells with TGFβ led to an increment of p-Smad2/3 that has a peak at 2 hours of TGFβ induction. On the other hand, cells treated with PRP did not show a significant increment in p-Smad2/3 after TGFβ stimulation in any of the time intervals tested (Fig. 5C,D).

We next studied the effect of PRP on the Smad-independent branch of TGFβ pathway by analyzing the phosphorylation of p38 after PRP treatment. Figure 5E (Supplementary Fig. S5) shows that the amount of phophoyilated p38 significantly decreased after treatment when compared with control cells.

Thus, PRP seams to act throught TGF-β patway, the inhibition of Smad2/3 and p38 phosphorylation may implay that these proteins will not be translocated into the nucleus to regulate multiple EMT-relevant transcription factors as is shown below.

### PRP inhibit EMT by directly enhancing E-cadherin and β-catenin expression

**Figure 6:**
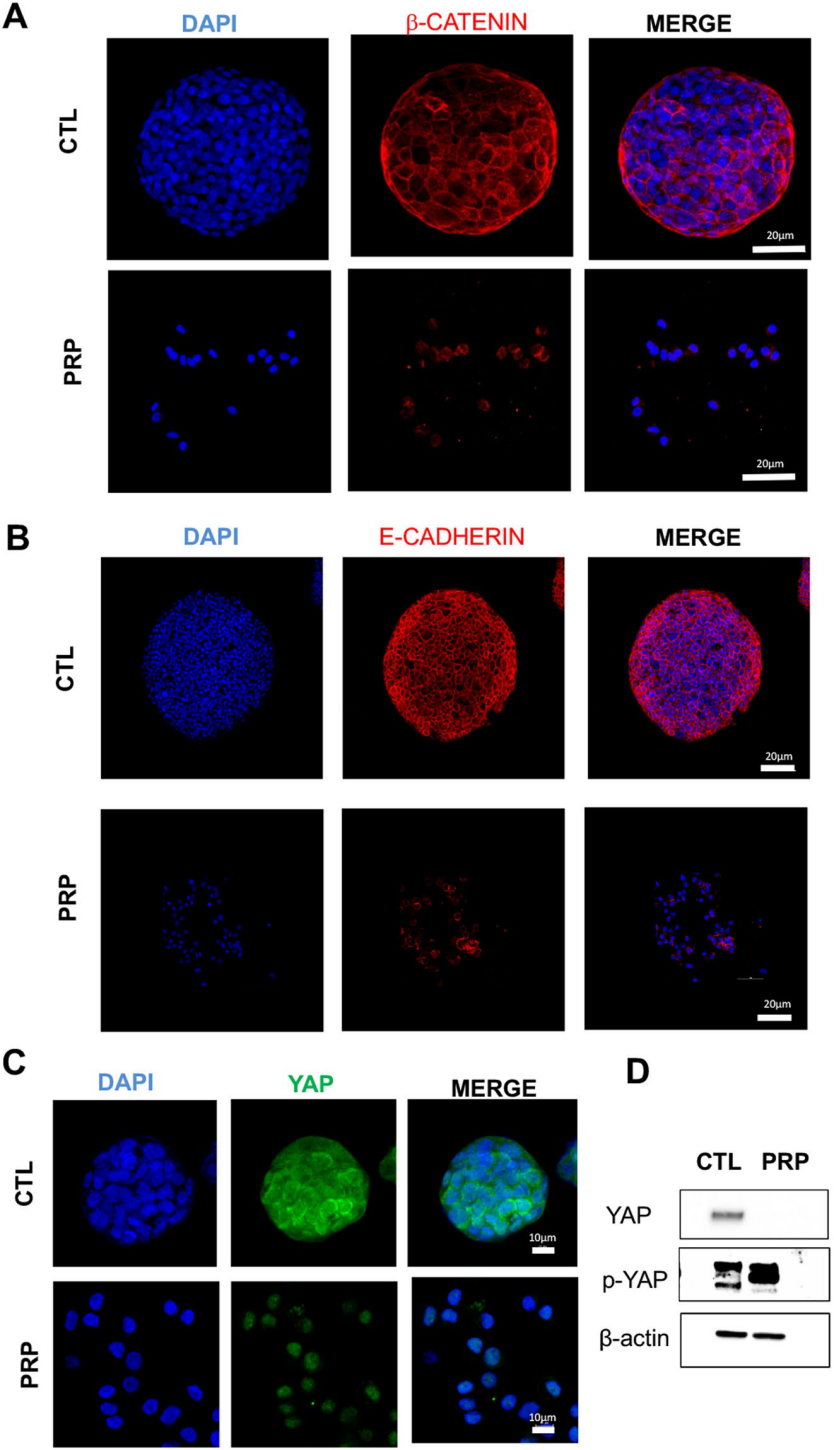
Representative confocal images of tumorigenic proteins in PRP treated pancreatic CSCs versus non-treated CSCs. (**A**) Treatment with PRP maintain membrane-associated β-catenin without nuclear translocation in pancreatic CSCs. (**B**) PRP treatment increased membrane-associated E-cadherin. Cell surface β-catenin and E-cadherin expression was detected in red and nuclei were counterstained with DAPI (blue). Scale bar represents 20 μm. (**C**) PRP treatment decreased the protein expression of YAP in BxPC3 CSCs. YAP expression appears green and nuclei stained with DAPI (blue). Scale bar represents 10 μm. (**D**) Western blot analysis of YAP and p-YAP proteins in CSCs treated with PRP versus non-treated. A negative correlation between YAP and p-YAP expression was found.

We performed immunological analyses to detect the expression of the cell adhesion markers β-catenin and E-cadherin. Our results show that non-treated CSCs displayed distinct membrane localization of β-catenin and weak, diffuse nuclear and cytoplasmic staining while PRP treated CSCs maintain membrane-associated and cytoplasmic staining of β-catenin without nuclear translocation (Fig. 6A). Treated cells maintained E-cadherin expression despite the PRP treatment destroyed the spheroid and many adhesion structures (Fig. 6B).

### Role of PRP in hippo pathway

To corroborate the hypothesis that PRP inhibits CSCs proliferation and tumorigenesis through the Hippo pathway, we further determined the impact of PRP on YAP and P-YAP expression. We found that PRP treatment tended to decrease the expression of YAP on CSCs as judged by immunofluorescence assay Fig. 6C. We then analyzed the expressions of YAP and p-YAP proteins by Western blotting. Analysis confirmed that PRP repressed YAP expression. Moreover, a negative correlation between YAP and p-YAP expression was found (Fig. 6D and Supplementary Fig. S6) with a marked increase in YAP phosphorylation induced by PRP treatment.

### PRP inhibits tumour initiation and growth on nude mice

**Figure 7:**
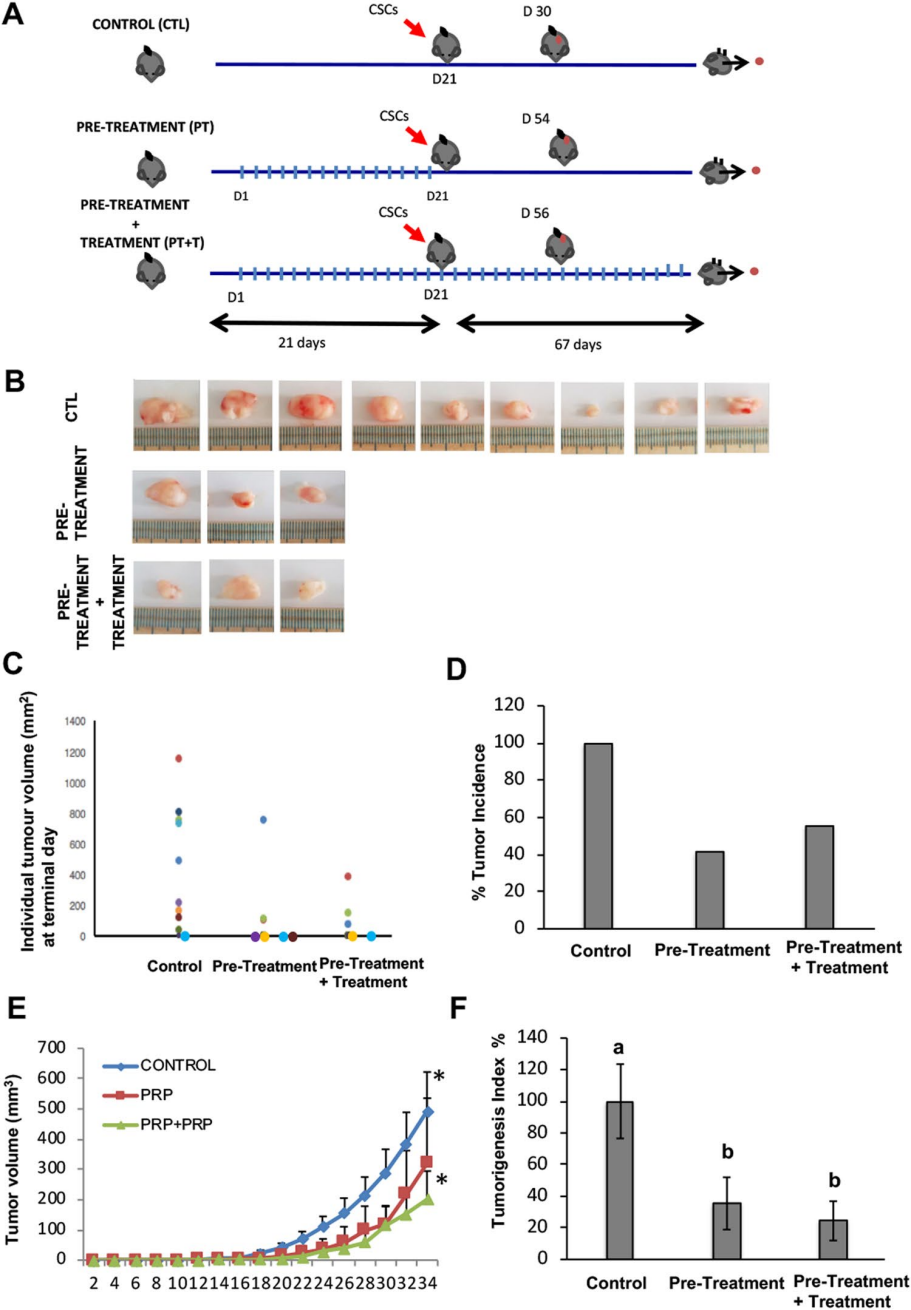
PRP reduced tumour growth of pancreatic CSCs *in vivo*. (**A**) Scheme of the *in vivo* study design to determine antitumour activity of PRP against pancreatic CSCs induced tumours. Perpendicular bars stand for PRP bolus injection. BxPC3 CSCs injection into the flank of nude mice is represented by a red arrow. (**B**) Photographs of the subcutaneous tumors isolated from mice included in the control and PRP treated groups at the end of the experimental protocol. (**C**) Tumour weight of each single tumour (different colour circles) at Termination (mg), mice with no tumour are represented by a colour circle on the X axis. (**D**) Tumour incidence. Percentage of mice with tumours respect to control group which represented a 100% of tumour incidence. (**E**) Tumour size evolution over time. MEM ± SE. *p < 0.05 vs. control group. (**F**) Tumorigenesis Index (TIn). This index relates tumour incidence and tumour weight. Percentage of mice with tumours respect to control group which represented a 100% of tumour incidence. Mean Tin ± SE at Termination. Different letters stand for significant differences (p < 0.05).

Here we show a xenograft study, with tumours generated from BxPC3 CSCs, to test the effect of PRP as a pre-treatment before tumour induction and as a pre-treatment and continuing treatment after tumour induction (Fig. 7A). 10 animals were used per group in agreement with others^17^. Nevertheless, one of the main difficulties of interpreting xenograft studies data is the loss of individuals, due to early death, that inevitably occurs during the time-period of the experiment^18^. In our study, 2 animals from the pre-treatment group and 4 animals from the pre-treatment + treatment group died before completing the experiment (due to anesthesia and ulceration issues). These sudden deaths were not related to an adverse effect of PRP, but rather due to technical problems with the compound administration. Results showed here have been normalized by the final “n” of each experimental group. We found that PRP treatment led to significant reduction in tumour development, thus 9 out of 10 control mice did present palpable tumours; on the contrary, only 3 out of 8 pre-treated mice and 3 out of 6 pre-treated + treated mice developed tumours (Fig. 7B,C). Therefore, tumour incidence (%) represented a 41% in the pre-treated mice and a 50% in the pre-treated + treatment group, indicating a clear suppressive effect of PRP on pancreatic CSC´s tumour engrafting (Fig. 7D). Further, the evolution of tumour growth was evaluated measuring tumour volumes every 2 days for 34 days (Fig. 7E), demonstrating that PRP treatment and the follow up treatment with PRP significantly inhibits tumour growth. Next, we examined the Tumourigenesis Index (TIn) that relates tumour incidence and tumour weight. This index can be used as a measure of malignancy or tumour aggressiveness. Figure 7F shows that significant differences were appreciated in TIn when the mice were pre-treated and treated with PRP, indicating the anti-tumourigenesis effect of PRP. In conclusion, PRP impaired engrafting of pancreatic CSC’s tumours in nude mice and displayed an antigrowth effect toward initiated xenografts.

**Figure 8:**
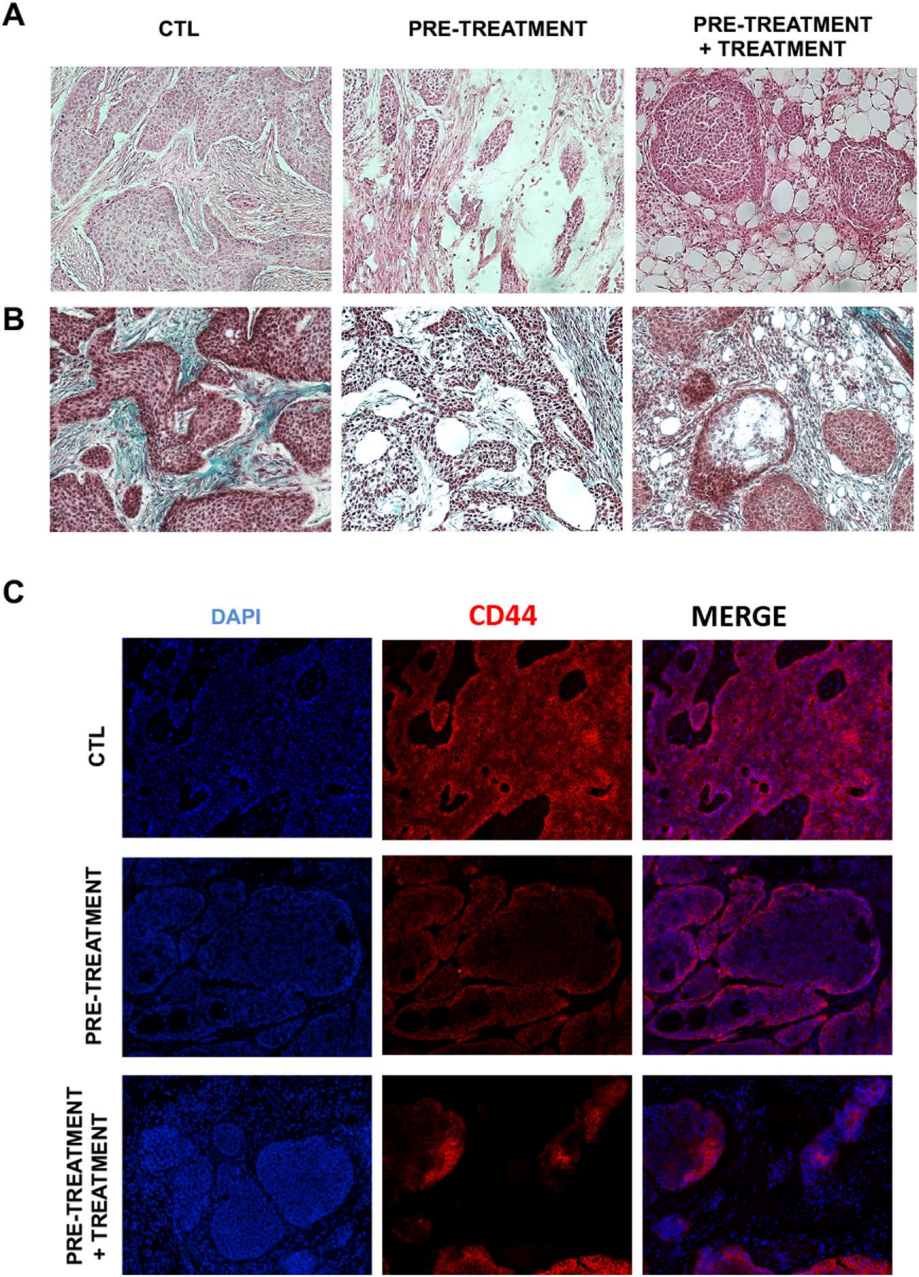
Effects of PRP on ECM deposition and pancreatic cancer marked CD44 expression. Representative images of tumour sections histologicaly staining with haematoxylin-eosin (H&E) (**A**) and Masson’s Trichrome (MS). (**B**) Tumours isolated from treated mice showed less fibrotic tissue (pink staining for the H&E test and green staining for the MS labellin) when compared with tumours isolated from control mice. (**C**) Immunofluorescence staining of pancreatic tumour sections with CD44 (red channel) demonstrated that tumours from non-treated mice had increased expression of CD44. Magnification, 10×.

The histological analysis of BxPC-3 tumours sections showed differences in tumour characteristics, particularly in the amount of fibrotic tissue, when comparing tumours of the same size. Large necrotic areas were observed (nude areas) in treated tumours compared to untreated controls (Fig. 8A,B). Hematoxilin and eosin staining showed an increased labelling of stromal proteins in control tumour tissues when compared to tumour sections from the pre-treatment and pre-treatment + treatment groups (Fig. 8A). In adittion, Masson’s Trichrome staining showed that pancreatic CSCs induced tumours form large islets of cohesive cells embedded in an abundant collagenous stroma (green) (Fig. 8B, CTL). Interestingly, the presence of extracellular matrix proteins fibers was strongly reduced in tumours isolated from mice that had been treated with PRP. The presence of abundant lacunae and unhealthy connective tissue can be seen within the fibrotic tissue area (Fig. 8B).

Moreover, the tumorigenic stem-like cell marker CD44 expression was analysed in tumour sections by immunofluorescence assay. Control tumour sections exhibited a strongly positive CD44 labelling while tumour sections bellonging to the treatment groups displayed weak CD44 staining consistent with the antitumour effect produced by PRP treatment (Fig. 8C).

## Discussion

Cancer stem cells (CSCs) have been characterized as immortal cells (due to their high telomerase activity) with a great tumour-initiating capacity. Furthermore, they can self-renew and they have unique properties, including pluripotent features, high metastatic potential, quiescent capacity and radio- and chemo-resistance^1^, thus strategies to eradicate CSCs population are needed to prevent cancer recurrence.

Here we have tested the potential therapeutic effect of a pro-enzyme pancreatic preparation, PRP, on pancreatic enriched CSCs population, and shown that PRP treatment *in vitro* decreased the percentage of ALDH1-positive cells as well as the percentages of cells expressing the pancreatic CSC-specific biomarkers CD326, CD44 and CXCR4^19^. In addition, PRP treatment induced an inhibition of both primary and secondary sphere formation ability of treated pancreatic CSCs, in fact, tumour-derived spheroid structures have been reported to be related to an enrichment of the CSC subpopulation^20^.

To determine the effect of PRP treatment at mRNA expression level we performed a series of microarray analysis and proved that a wide range of genes closely related to the CSCs phenotype, the EMT process, cell proliferation, metastasis, cell adhesion, tumour suppression mechanisms, cytokine signalling and kinase activities were altered by the treatment. We found that OCT4 which has been reported to play a key role in the maintenance of a stem-like phenotype in pancreatic CSCs was down-regulated by PRP^21^. Moreover, it is important to highlight the down-regulation of DLK1, induced by PRP treatment, as this factor has been reported to play a significant role in the ability to form spheroid-like structures associated with the CSC phenotype^22^. These results correlate with the decrease in the CSCs population after PRP treatment shown above.

In addition, PRP treatment induced up-regulation of proven tumour suppressor genes such as the transcription factor FOXP1, a tumour suppressor lost in several tumour types^23^. and TWIST2 and THY1^24,25^.

Interestingly, the Notch signalling pathway, which is aberrantly activated in many types of malignancies and which is a key regulator of the EMT process^26,27^, was found to be inhibited by PRP. In fact, the expression of several Notch initiating genes, including NOTCH1, JAG1, FGF and PDGFR was downregulated by PRP treatment. Furtheremore, EMT-related genes, such as Snai1 and Snai2 (also known as Slug) that are target genes of the Notch pathway were down-regulated by PRP treatment. In addition, E-cadherin, a gene suppressed by the Notch pathway, was up-regulated after PRP treatment^26^.

In studying cytokine expression we found that interleukin-8 (IL-8), a pro-inflammatory factor, belonging to the CXC chemokine family was down-regulated by PRP. Cancer cells secrete IL-8 in an autocrine manner to achieve a proper tumour microenvironment^28,29^. Particularly, IL-8 and IL-8 receptors have been shown to be over-expressed in pancreatic cancer, but suppressed in normal pancreatic tissues, suggesting that IL-8 plays an important role in the invasiveness of human pancreatic cancer^30^. In addition, treatment with PRP dramatically decrease the expression of SPP1. This protein plays a crucial role in determining the metastatic potential of various cancers^31^, promotes cancer cell survival and regulates tumour-associated angiogenesis and inflammation^32^. Moreover, both JNK and ERK pathways were down-regulated as a consequence of PRP treatment, which is significant since those pathways are crucial in the control of cell proliferation and tumour malignancy. We observed a marked down-regulation of MEK1 (MAP2K1) a cue member of the ERK pathway. Remarkably, MEK inhibitors have been tested in clinical trials as anti-tumour agents, for instance PD0325901, a second generation MEK1/2 inhibitor^33^. Therefore, PRP seems to control cell proliferation by inhibiting both JNK and ERK pathways through suppression of the expression of two relevant oncogenic transcription factors JUN and FOS.

Furthermore, PRP induced the up-regulation of cyclin-dependent kinase inhibitors genes (CDKN2A; CDKN2, 2 and CDKN2C) which are considered important tumour suppressor genes. Here we found that PRP significantly decreased the expression of DLK1 in treated pancreatic CSCs. Recent studies have shown that DLK1 knockdown could suppress the ability of proliferation, colony formation, spheroid colony formation, and *in vivo* tumorigenicity of hepatic CSCs^22^. In addition, the SFN-1 (YWHAZ) gene was dramatically down regulated by PRP treatment. YWHAZ knockdown by specific siRNAs, has been shown to inhibit the proliferation, migration, and invasion of YWHAZ-overexpressing gastric cancer cells^34^. Feldmann and Maitra (2008)^35^ have reviewed the molecular genetics of pancreatic ductal adenocarcinomas and presented three important tumour suppressor genes that are deleted in a high percentage of pancreatic cancers: p16; SMAD4 and TP53. These genes inhibit cell proliferation by arresting cell cycle progression. Of interest, PRP up-regulates the expression of these same genes, suggesting the role of PRP in suppressing cell proliferation and malignant transformation.

Some studies state that the TGFβ signalling pathway can exert a tumour suppressive role in earlier stages of tumour development and change to a tumorigenic pathway in the advanced stages after losing Smad4^36^, TGFβR2^37^, TGFβR3^38^ or RAC1b^39^ expression and gain overexpression of TGFβ1^39^. Interestingly, our results reveal a down-regulation of TGFβ1 and an up-regulation of TGFβ2, TGFβ3, Smad4 and RAC1b in treated cells, suggesting that PRP treatment promotes the TGFβ pathway role via a tumour suppressive route.

In the line of the changing nature of TGFβ pathway during tumour progression, it has been pointed that Smad7, a TGFβ pathway component, can be considered as a “swith” acting as an inhibitor of TGFβ pathway in early stages of tumour progression and as a enhancer of tumour invasion in late stages. Indeed, Smad7 has been shown to be overexpressed in many cancer types and its abundance can be positively correlated with the malignancy potential^40^. Here we shown a down-regulation of Smad7 in treated cells, supporting the idea that PRP treatment promotes a deep change in TGFβ pathway and decreases tumour malignancy. Additionally, PRP treatment up-regulated KLF17 expression, which enhances TGFβ/Smad-dependent signalling pathway directing cell differentiation^41^ and not toward cell stemness. Moreover, treated pancreatic CSCs exhibited a decreased activation of both Smad-dependent and Smad-independent branches of TGFβ pathway as shown by the reduction Smad2/3 phosphorylation, even under TGFβ stimulation, and the reduction of p38 phosphorylation. Interestingly, RAC1b has been described to act as a “retaining wall” of TGFβ pathway preventing the hyperactivation of Smad-dependent and Smad-independent branches of TGFβ pathway^42,43^. Here we have shown that PRP up-regulate RAC1b which matches with the decreased phosphorylation of Smad2/3 and p38 found.

The expression of miR-21-5p, miR-182-3p and miR-7 was analysed after PRP treatment and was found that PRP treatment decreased the expression of miR-21-5p and miR-182-3p and up-regulated the expression of miR-7. FOS and JUN form the protein complex known as Activated Protein 1 (AP1) which plays a key role in cancer development^44^ and is up-regulated in the CSCs population promoting chemoresistance through miR-21 enhancement in these cells^45^. Therefore, the down-regulation of FOS and JUN and the down-regulation of miR-21 suggests that PRP treatment may inhibit the activity of AP-1 complex and, subsequently, it may promote the chemo-sensitization of the treated pancreatic CSCs, which is in agreement with the disruption of the CSC phenotype discussed above. Furthermore, miR-21 has been described to suppress Smad4 expression cells^46^, thus the down-regulation of miR-21 after PRP treatment seems to be linked to the up-regulation of Smad4 and to the alteration of the TGFβ pathway found in treated cells. PRP treatment also diminished the expression of miR-182-3p, others have proposed that up-regulation of miR-182 correlates with the hyperactivation of TGFβ pathway^47^, therefore, the down-regulation of miR-182 in treated CSCs supports the hypothesis that PRP may be suppressing the hyperactivation of TGFβ pathway. Last, PRP treatment up-regulated the expression of miR-7 which is considered a tumour suppressor since it has been found to suppress the EMT process and the stemness of CSCs in some tumours^48,49^.

Moreover, mRNA levels of MST1/2, SAV1 and LATS1/2 were found to be upregulated by PRP, together with an increased amount of phospho-YAP protein proved by western blotting. YAP and TAZ belong to the Hippo signalling pathway and are accumulated in the cytoplasm by phosphorylation prompted by MST1/2, SAV1 and LATS1/2. Thus, phosphorylation secuestrates these proteins in the cytoplasm, preventing migration to the nucleus and the activation of downstream genes involved in cell proliferation^50,51^. Likewise, the inhibition of the activation (phosphorylation) of p38 shown here is relevant since p38 pathway has been described as blocking the phosphorylation of YAP, which correlates with the increment of phosphorylated YAP shown here after PRP treatment of pancreatic CSCs^52^. It appears that PRP treatment inhibits the uncontrolled proliferation promoted by an aberrant hyperactivation (dephosphorylation) of YAP, which is a common event in many types of malignancies^51,53,54^.

In addition, cytoplasmic phospho-YAP has been reported to promote the phosphorylation and the retention of β-catenin in the cytoplasm^55^, which correlates with our findings with immunofluorescent assays. β-catenin is a key component of the Wnt signalling pathway, which is over-activated causing a strong enhancement of cell proliferation and tumour progression in many types of malignancies^56^. In the absence of Wnt-related ligands (Wnt1, Wnt2, Wnt3…), β-catenin is consistently phosphorylated and retained in the cytoplasm and, subsequently, it cannot migrate to the nucleus and activate pro-tumorigenic target genes^57^. According to these data along with the down-regulation of some Wnt-related initiating ligands such as WNT1 or WNT11 and the up-regulation of several Wnt pathway-suppressors including SOX10^58^ and SIRT1^59^ shown here, we conclude that PRP treatment strongly inhibits the canonical WYT pathway^56^. Adittionaly, the increased phosphorylation of YAP after PRP treatment could also explain the inhibition of Notch pathway suggested above^54^.

Finally, we present a xenograft study to analyse the effect of PRP *in vivo*. Our results suggest that pre-treatment with PRP might inhibit the production and the action of growth factors and angiogenic stimulating factors that are necessary to prepare the tumour niche and, consequently, it reduces the possibility of tumour engrafting. In fact, the anti-angiogenic effect of PRP has been previously proven by our previous studies^9^. Interestingly, TGFβ-1, an essential factor to create, maintain and support CSCs^60^ was strongly down-regulated after PRP treatment, which agrees with other studies that demonstrated oral therapy with proteolytic enzymes decreased TGFβ in human blood (Desser *et al*. 2001).

Moreover, our histological analysis of the xenografting mice models shows a decreased amount of tumour-surrounding fibrotic tissue of treated mice which can be explained by the strong down-regulation of TGFβ-1 by PRP treatment as TGFβ-1 has been described to be closely related to the corruption of healthy fibroblasts into cancer-associated fibroblasts (CAFs)^61,62^. Additionally, it has also been reported that the surrounding tumour microenverioment also promotes the appearance of new CSCs derived from non-stem cancerous cells by secreting several factors such as IL6, HGF or TGFβ-1^63^. Consequently, pre-treatment and follow up treatment with PRP might decrease TGFβ-1 and, subsequently, it might impair CSCs tumour engrafting, niche formation and even CSCs subpopulation activation. Further, treated tumours showed a decreased expression of the pancreatic cancer marker CD44. Knockdown of CD44 expression in pancreatic cancer cells has been linked with decreased cellular proliferation, invasiveness and increased sensitivity to gemcitabine^64^.

In summary, the present study demonstrates the high anti-tumour efficacy of PRP against human pancreatic CSCs, both *in vitro* and *in vivo*. The molecular PRP findings presented demonstrates that PRP treatment promotes the up-regulation of RAC1b which avoids the hyper-activation of the p38 pathway induced by the TGFβ pathway leading to the phosphorylation of YAP observed in our results, which also explains the blockage of the canonical Wnt pathway, the inhibition of the Notch pathway as well as the cytoplasmic location of β-catenin in treated cells as discussed above. Morover, the regulation of TGFβ, Hippo, Wnt and Notch pathways could explain the disruption of the CSC phenotype and the EMT process found in treated cells. Finally, we reported that PRP impaired engrafting of pancreatic CSC’s tumors in nude mice and displayed an antigrowth effect toward initiated xenografts, making (pro)enzymes treatment a valuable strategy to reduce CSC population for pancreatic cancer therapy. As cancer treatment moves towards more personalised medicine, proper therapies to target and treat specifically CSCs may prove to be a useful method for reducing recurrence after drug treatment failures.

## Material and Methods

### Pancreatic pro-enzymes

The pancreatic pro-enzymes Chymotrypsinogen A and Trypsinogen were supplied by AplyChem. A stock solution of 6 mg/ml of Chymotrypsinogen A and 1 mg/ml of Trypsinogen was made dissolving the pancreatic enzymes in PBS and stored at −20 °C. For each experiment, the stock solution was further diluted in a medium to obtain the desired concentrations.

### Cell line

The human pancreatic cancer cell lines BxPC3 was obtained from American Type Culture Collection (ATCC) and maintained in RPMI 1640 Medium (Sigma-Aldrich) supplemented with 10% FBS. Cell disassociation for cell passing was done with the trypsin replacement TrypLE reagent (Life Technologies).

### Isolation of CSCs

Cancer stem-like cells from BxPC3 pancreatic cancer (adenocarcinoma) cell line were isolated using the ALDEFLUOR assay (StemCell Technologies) by fluorescence-activated cell sorting (FACS) and enriched subpopulation of CSCs were grown with our specific sphere forming medium (PCT/ES2015/070606, Jiménez *et al*. 2018) in ultralow attachment plates (Corning).

### MTT cell proliferation assay

The effect of PRP on cell viability and proliferation was assessed using the MTT (3-(4, 5-dimethylthiazolyl-2)-2, 5-diphenyltetrazolium bromide) assay (Sigma-Aldrich). Briefly, cells (2 × 10^3^ cells/well) were seeded onto 96- well plates and incubated for 24 h and then treated with different PRP concentrations (Fig. 1A). Three days later, treatment was repeated and cells were maintained for 3 additional days. Cells were maintained with the drug for six days. Thereafter, cells were processed as follows, 10 μL of 2 mM MTT reagent was added to each well and cells were incubated at 37 °C for 4 hours. 100 μL detergent reagent was then added and cells were left at room temperature in the dark for 2 hours. Absorbance was recorded at 570 nm. The IC50 values were calculated from four parametric logistic curves by linear interpolation using Sigma Plot software. All of the experiments were plated in triplicate wells and were carried out at least twice.

### ALDEFLUOR assay and CSCs markers analysis by flow cytometry

Cancer stem-like cells from BxPC3 cancer cell line were labelled using the ALDEFLUOR assay kit according to the manufacturer’s recommendations (StemCell Technologies). The enriched subpopulation of CSCs was characterized using a FACSAria III flow cytometer (Becton Dickinson).

To analyse CSC markers treated and not treated BxPC3 CSCs were disaggregated by tryple and washed twice in PBS supplemented with 1% bovine serum albumin (Sigma-Aldrich). The cell surface Fc receptor was blocked using IgG (Santa Cruz Biotechnology) on ice for 15 min. Cells were stained for 30 min at 4 °C with anti-CD44-PE and anti-CD326 FITC and CxCR4-APC monoclonal antibodies (BD Biosciences). After washing, cells were analyzed using a FACSAria III flow cytometer (Becton Dickinson).

### Effects of PRP and gemcitabine on BXPC-3 CSCs proliferation

Enriched subpopulations of CSCs from formed primary and secondary spheres were seeded in a concentration of 3000 cells/well in 96-well plates in sphere forming medium and treated with PRP (T/C 0.07/0.42 mg/mL), gemcitabine at 0,01 μm and with both PRP (T/C 0.07/0.42 mg/mL) and gemcitabine 0,01 μm. After 72 h, 0.01 ml of Cell Counting Kit-8 (CCK-8) (Dojindo Molecular Technologies), was added to each well and incubated at 37 °C for 2 h. Plates were read at 450 nm on a Bio-Rad plate reader.

### PCR microarray analysis of human EMT, CSCs and MAP kinase signalling pathway related genes

BxPC3 enriched CSCs were grown in 6 well plates and treated twice with PRP (T/C 0.07/0.42 mg/mL) on day 2 and on day 4 or non-treated control cells (CTL). On day 5, total cellular RNA was isolated using TRIzol reagent (Life Technologies). A pool of three total RNAs extracted from three independent experiments was used for first strand cDNA synthesis using the kit SuperScript IV First-Strand Synthesis System (ThermoFisher Scientific).

PRP induced changes in expression of genes involved in the EMT process, related to CSCs or involved in MAP Kianse Signalling Pathway were analysed with RT^2^ Profiler PCR Arrays Human Epithelial to Mesenchymal Transition (PAHS-090Z, SABiosciences); RT^2^ Profiler PCR Arrays Human Cancer Stem cells (PAHS-176Z, SABiosciences) or RT^2^ Profiler PCR Arrays Human MAP Kinane Signalling Pathway (PAHS-176Z, SABiosciences) respectively, and processed following the manufacturer’s instructions applied on the ViiA7 fast real-time PCR system (Applied Biosystemsen). A set of controls present on each array enabled data analysis using the ΔΔCT method of relative quantification and assessment of reverse transcription performance, genomic DNA contamination, and PCR performance.

Data analyses were performed using the web-based analysis software (http://pcrdataanalysis.sabiosciences.com/pcr/arrayanalysis.php).

### *In vivo* anti-tumour xenograft studies

To establish xenograft tumours six- to eight-week-old NSG immunodeficient mice were used. All procedures were approved and performed in accordance with the guidelines of the Institutional Animal Care and the Research Ethics Committee of the University of Granada. Mice were housed and maintained at 20 °C to 24 °C, 50% RH, a 14 to 10 h lightdark cycle with food and water ad libitum.

Animals (n = 10 per group) were randomly assigned as control, pre-treatment group and pre-treatment + treatment group. Treated groups received Trypsinogen and Chymotrypsinogen A at a dose of 83.3/500 mg/kg in combination in a single injection administered 3 days per week via intravenous tail vein injection in a dosing volume of 10 mL/kg. The volume of dosing solution administered to each animal was calculated and adjusted based on individual body weight measured immediately prior to dosing. Treatments were administered for 3 weeks before tumour induction (pre-treatment group) and for 3 weeks before tumour induction and continued during 9,5 weeks after tumour induction (pre-treatment + treatment group); control group was inoculated with a physiological saline solution (Fig. 7A).

Tumours were generated by subcutaneous injections of BxPC3 CSCs, to do so, CSCs spheres were disaggregated and 500 viable cells were injected per mouse using 26-gauge needles. Tumour size was calculated according to the formula:

$${\rm{tumour}}\,{\rm{volume}}\,{(\mathrm{mm}}^{3})={{\rm{d}}}^{2}\times {\rm{D}}/2,$$

where d and D are the shortest and longest diameters, respectively. Paraffin-embedded blocks of all tumours were sectioned at 5 μm. Each sample was stained with hematoxylin and eosin (H&E) and Mason tricrome for histopathologic analysis. For CD44 staining tumour sections were labelled with HCAM (DF1485) (sc-7297, Sigma-Aldrich), at 4 °C overnight. Following washing with PBS, tumour sections were subsequently incubated with fluorescence conjugated secondary antibody for 2 h at RT. The sections were then mounted with mounting medium containing DAPI (Vector Laboratories). For negative controls, primary antibodies were replaced with PBS. Images were captured using a confocal fluorescence microscope (Leica DM 5500B microscope).

### Quantitative real time RT-PCR

ALDEFLUOR sorted BxPC3 pancreatic cancer cells were maintained for 6 days in culture and treated twice (day 2 and day 5) with PRP - Chymotrypsinogen: 0.42 mg/ml - Trypsinogen: 0.07 mg/ml. Total RNA was extracted using the TRIZOL reagent following the instructions of the manufacturer (Life Technologies). cDNA was synthesized by reverse transcription of total RNA using the Reverse Transcription System (Promega) and qRT-PCR assay was done using SYBR Green PCR Master Mix (Promega) and random primers. Each reaction was performed in triplicate from two cDNA dilutions. The comparative threshold cycle (Ct) method was used to calculate the amplification factor as specified by the manufacturer. Human GADPH was used as an internal standard to normalize variations in RNA quality in the quantities of input cDNA. The following primers were used for detection of Rac1 + Rac1b: 5′-ACCATGCAGGCCATCAAGTGTGTGG-3′ and 5′-TTACAACAGCAGGCATTTTCTCTTC-3′. Measurement of Rac1b was performed with exon 3b-specific primers: 5′-GGAGAAACGTACGGTAAGGATATAACC-3′ and 5′-GGCAATCGGCTTGTCTTTGCCC-3′. Smad7 primer was obtained from Sigma. Primer sequences used for GAPDH was: 5′-TGCACCACCAACTGCTTAGC-3′ 5′-GGCATGGACTGTGGTCATGAG-3′.

### miRNA real-time reverse transcriptase PCR

To determine the expression of miRNAs (miR-21, miR-182, miRNA-7 and miR-SNORD 44) (Exiqon) in control non-treated cells/PRP treated cells, TRIZOL Reagent (Life Technologies) was used according to the manufacturer’s recommendations. Reverse transcription from miRs was performed using the miRCURY LNATM Synthesis kit II (Exiqon) following the manufacturer’s protocol. qPCR was performed using miRCURY LNA TM EXILENT SYBR Green (Exiqon) in a CFX96 real-time PCR detection system (Bio-Rad). Each reaction was performed in triplicate and the comparative threshold cycle (Ct) method was used to calculate the amplification factor. Human Snord 44 was used as housekeeping.

### TGFβ stimulation

BxPC3 enriched CSCs were grown in 6 well plates and treated twice with PRP (T/C 0.07/0.42 mg/mL) on day 2 and on day 4 or non-treated control cells (CTL). On day 5, Recombinant Human TGF-beta 1 Protein (R & D Systems) was added to the cells in a concentration of 5 ng/ml, after 2, 4 and 10 hours of TGFβ stimulation cells were collected and processed for western blotting analysis.

### Western blotting

Whole cell lysates were obtained through RIPA lysis buffer (Santa Cruz Biotechnology). The cytosolic and nuclear proteins were extracted with the use of a nuclear protein extraction kit (Beyotime Biotechnology). Protein concentrations were then determined using Bio Rad detergent compatible protein assays (Bio Rad Laboratories). Equal amounts of protein were loaded onto on SDS–PAGE gel, transferred onto nitrocellulose membranes (Bio Rad,162- 0115), and blocked in PBS containing 5% non-fat dry milk for 1 h at room temperature. Primary antibodies used included HCAM (DF1485) (sc-7297, Sigma-Aldrich), CXCR-4 (4G10) (sc-53534, Santa Cruz), Ep-CAM (CD326) (EBA-1) (sc-66020, Santa Cruz), Phospho-Smad2/3 (pThr8, MERCK), Smad2/3 Antibody (07–408, MERCK), Phospho-p38 MAPK ((Thr180/Tyr182), D3F9), (#4511, Cell SignalingTechnology), p38 MAPK Antibody (#9212, Cell SignalingTechnology), GAPDH (#2118, Cell SignalingTechnology), Anti-Yap1(phosphoS127) antibody (ab76252, Abcam), Anti-YAP1 antibody [EP1674Y] (ab52771, Abcam) and β-ACTIN (A2228, Sigma-Aldrich) Secondary antibodies used included anti-rabbit IgG peroxidase conjugate (A0545, Sigma-Aldrich) and anti-mouse IgG peroxidase conjugate (A9044, Sigma-Aldrich). Protein–antibody complexes were visualized by enhanced chemiluminescence (ECL, Bonus) on a LAS 3000 Imaging System (Fujifilm). Protein band Intensities were quantitated using Image-J software (NIH).

### Immunofluorescence analysis

Cells were fixed with 4% paraformaldehyde in PBS for 20 min at room temperature (RT), blocked for 1 h at room temperature with 5% BSA, 5% FBS in PBS and incubated with the primary antibody overnight at 4 °C. Primary antibodies used were: HCAM (DF1485) (sc-7297, Sigma-Aldrich), CXCR-4 (4G10) (sc-53534, Santa Cruz), Ep-CAM (CD326) (EBA-1) (sc-66020, Santa Cruz), β-catenin (E-5) (SC-7963, Santa Cruz), E-cadherin (67A4) (SC-21791, Santa Cruz), Sox-2 (H-65) (sc-20088, Santa Cruz) Anti-YAP1 antibody [EP1674Y] (ab52771, Abcam) or p-Smad2/3 (SC11769, Santa Cruz).The next day, samples were washed three times with PBS, incubated with the secondary antibodies (Alexa) for 1 h at RT, washed three times with PBS and mounted with 4′,6-diamidino-2-phenylindole (DAPI)-containing mounting medium. Images were acquired with a Leica DM 5500B microscope.

## Supplementary information

Supplementary Data
