# Supplementary material for: Pancreatic (pro)enzymes treatment suppresses BXPC-3 pancreatic Cancer Stem Cell subpopulation and impairs tumour engrafting
Source: Sci Rep. 2019 Aug 6;9:11359. doi: 10.1038/s41598-019-47837-7 (PMC6684636; doi:10.1038/s41598-019-47837-7)
Supplement: Supplementary file 1 — Supplementary Data [file 41598_2019_47837_MOESM1_ESM.pdf]

Supplementary Data

Pancreatic (pro)enzymes treatment suppresses pancreatic Cancer Stem Cell subpopulation and impairs tumour engrafting

Pablo Hernández-Camarero[1,2,6*], Elena López-Ruiz[1,2,3,6*], Carmen Griñán-Lisón[2,3,4,6], María Ángel García[2,3,5,6], Carlos Chocarro-Wrona[2,3,4,6], Juan Antonio Marchal[2,3,4,6], Julian Kenyon[7], Macarena Perán[1,2,6]

Contents

Fig. S1 Raw images of western blots.

Fig. S2 Profiler PCR Array of EMT and CSCs

Fig. S3 Raw images of western blots.

Fig. S4 Raw images of western blots.

Fig. S5 Raw images of western blots.

Fig. S6 Raw images of western blots.

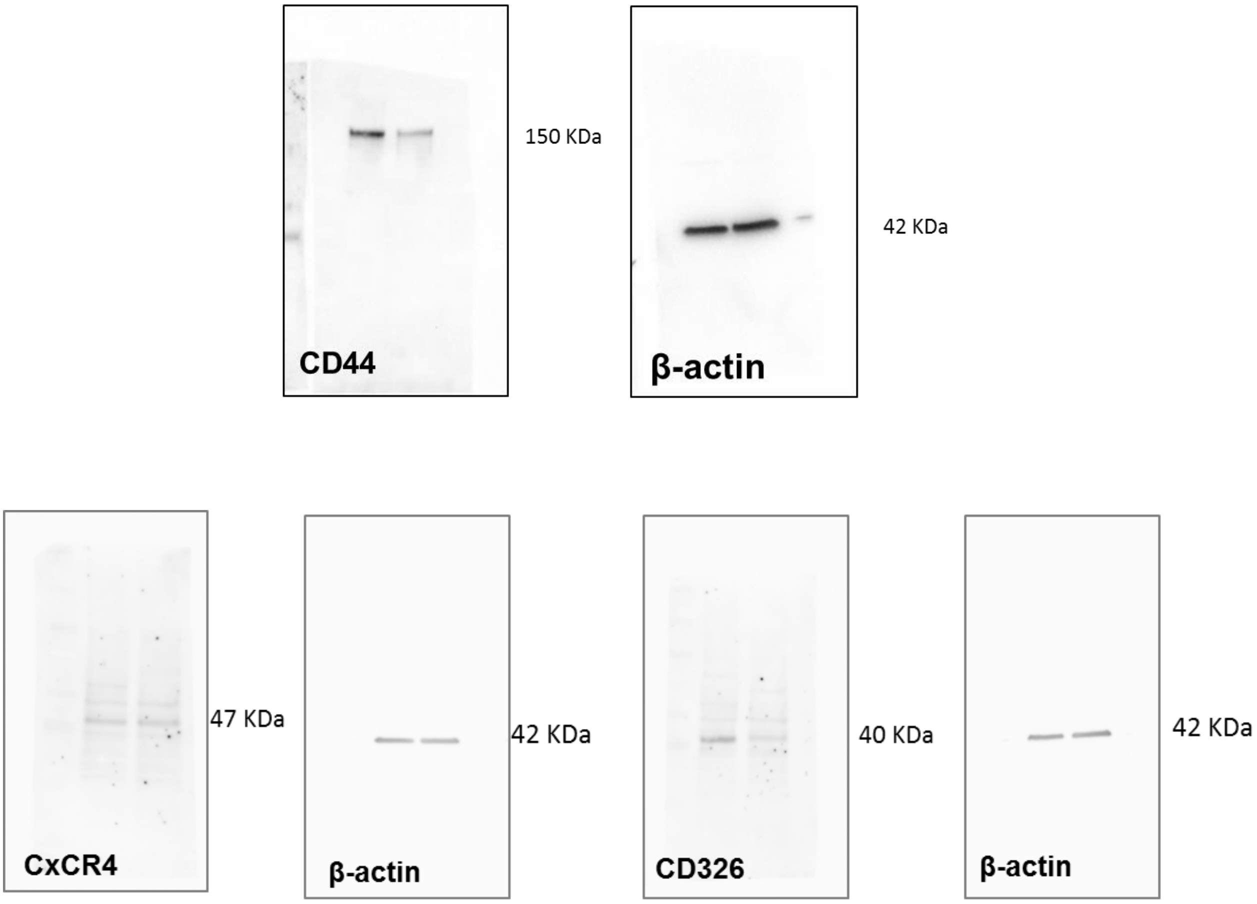

Fig. S1 Raw blots of western blots from Fig. 1D.

A

EMT

Genes Up-regulated

CAMK2N1	2,7
COL5A2	6,34
CTNNB1	4
ERBB3	2,73
FZD7	2,31
KRT7	3,67
MMP9	3,4
NODAL	4,7
NUDT13	2,37
DESI1	2,79
SOX10	4,9
SPARC	3,61
STEAP1	3,45
TFPI2	4,43
TGFB2	2,17
TGFB3	5,36
VCAN	2,06
VPS13A	2,22
ZEB1	2,89

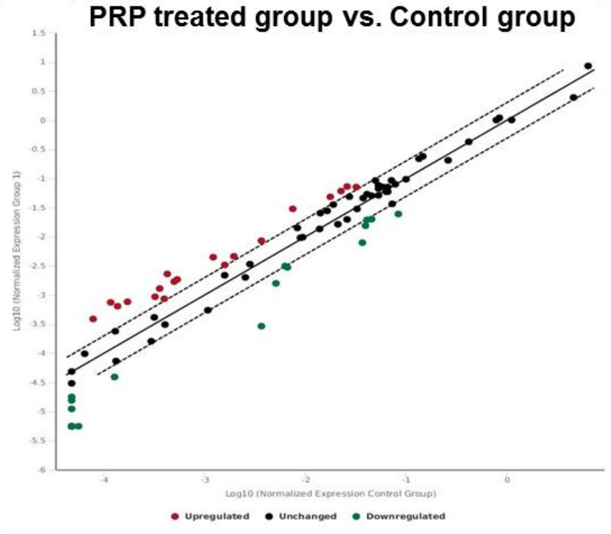

Genes Down-regulated

AHNAK	-8,64
CDH2	-8,64
COL1A2	-8,64
COL3A1	-3,3
ESR1	-8,64
FGFBP1	-2,27
FN1	-3,22
GNG11	-8,64
GSC	-8,64
IGFBP4	-2,71
IL1RN	-2,22
ITGA5	-2,57
JAG1	-2,11
KRT14	-12,64
MMP3	-8,64
NOTCH1	-2,01
PDGFRB	-3,11
SERPINE1	-4,66
SPP1	-10,14
TSPAN13	-3,45
WNT11	-4,36
ZEB2	-8,64

B

CSC

Genes Up-regulated

ALCAM	2,67
ATM	2,05
AXL	2,73
CHEK1	2,07
EGF	8,91
ENG	2,13
FGFR2	2,15
FOXP1	6,04
KLF17	5,96
LATS1	2,1
LIN28A	2,44
MAML1	2,11
MUC1	3,55
MYC	2,87
NANOG	23,52
NOTCH2	2,31
SAV1	2,71
SIRT1	2,07
SOX2	16,31
TGFBR1	2,1
THY1	3,62
TWIST2	5,27
ZEB1	4,69
ZEB2	4,11

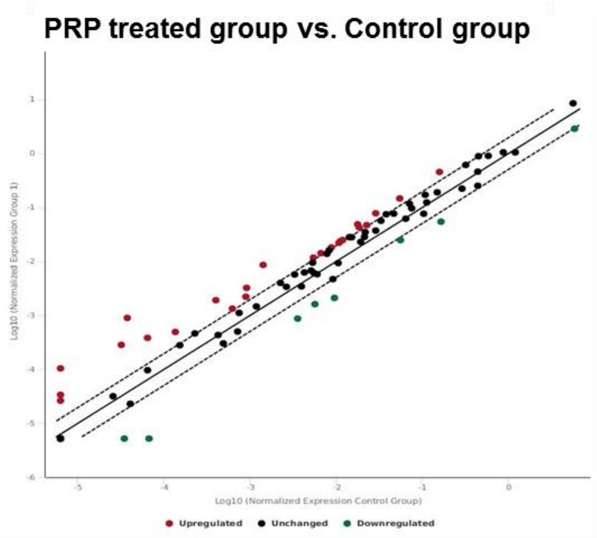

Genes Down-regulated

CD38	-12,82
IKBKB	-3,54
CXCL8	-4,56
JAG1	-2,26
PLAUR	-3,02
PTPRC	-6,72
SNAI1	-4,16
GAPDH	-2,04

Fig. S2. BxPC3 CSCs were treated with PRP on day 2 and on day 4. On day 5, total

RNA from treated and non-treated CSCs was extracted. A pool of three total RNAs extracted from three independent experiments was used for first strand cDNA synthesis. Gene expression was determined using RT2 Profiler PCR Array of EMT and CSCs. The scatter plots show all the genes that were up or down -regulated after treatment with PRP.

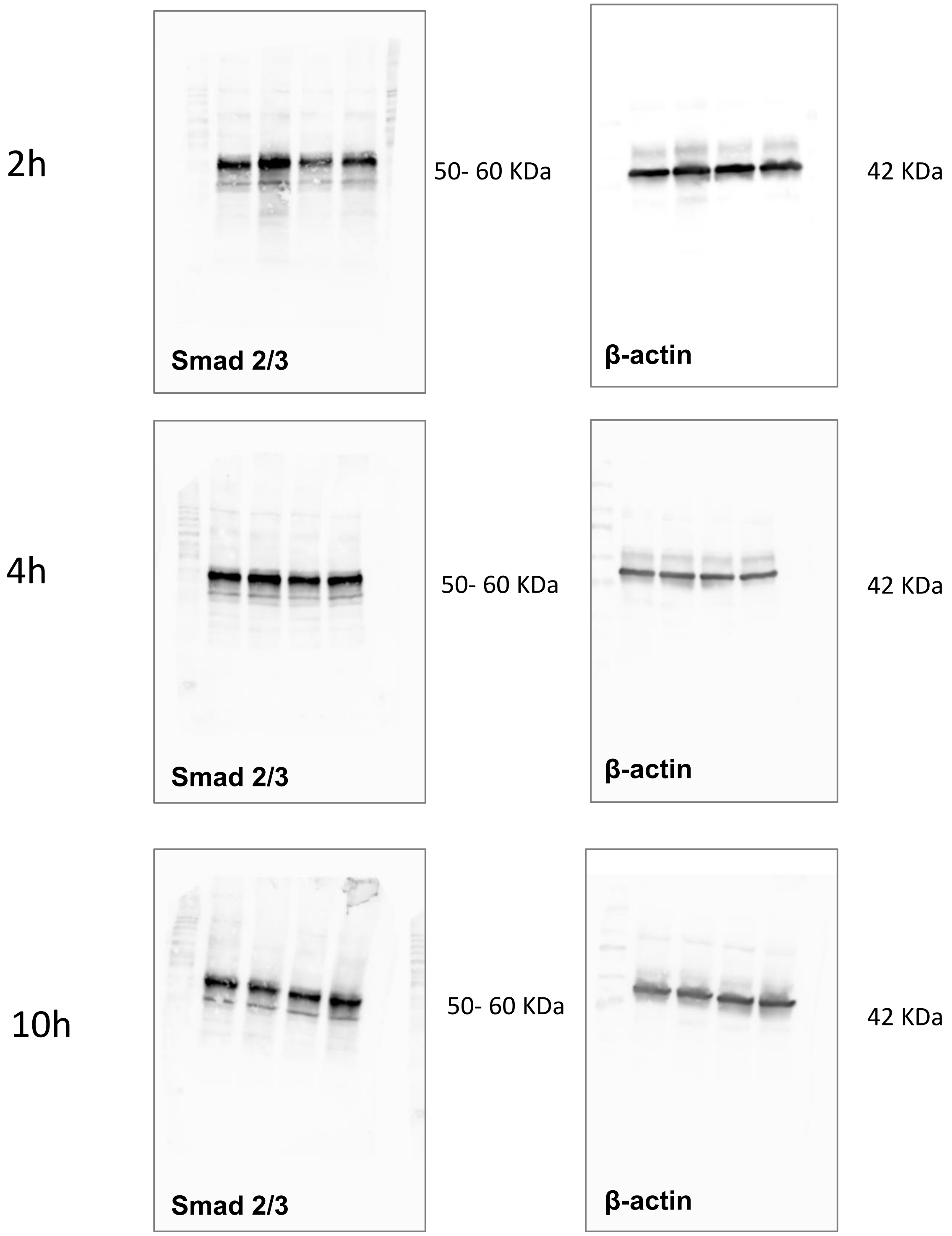

Fig. S3. Raw blots of western blots from Fig. 5C

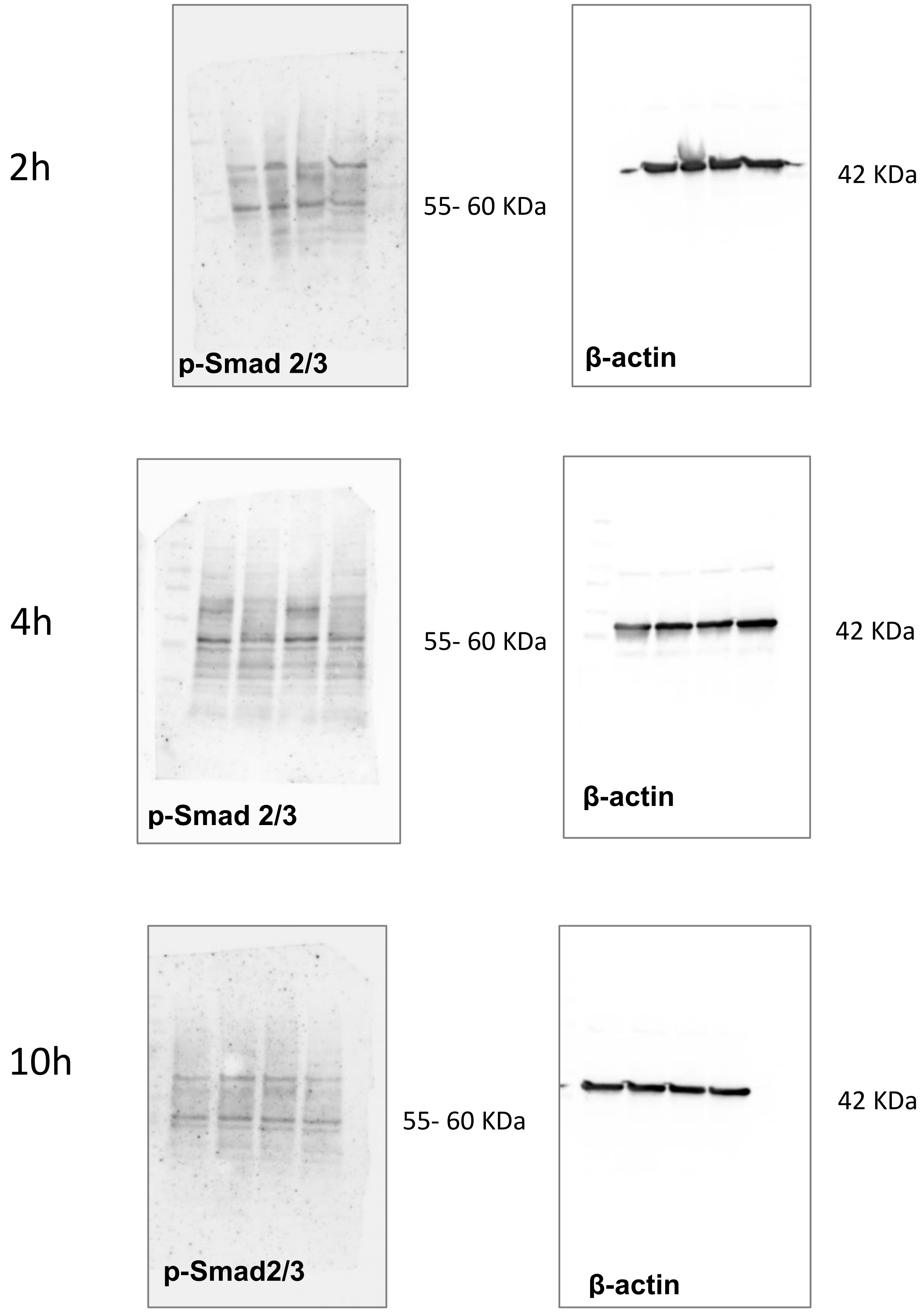

Fig. S4. Raw blots of western blots from Fig. 5C

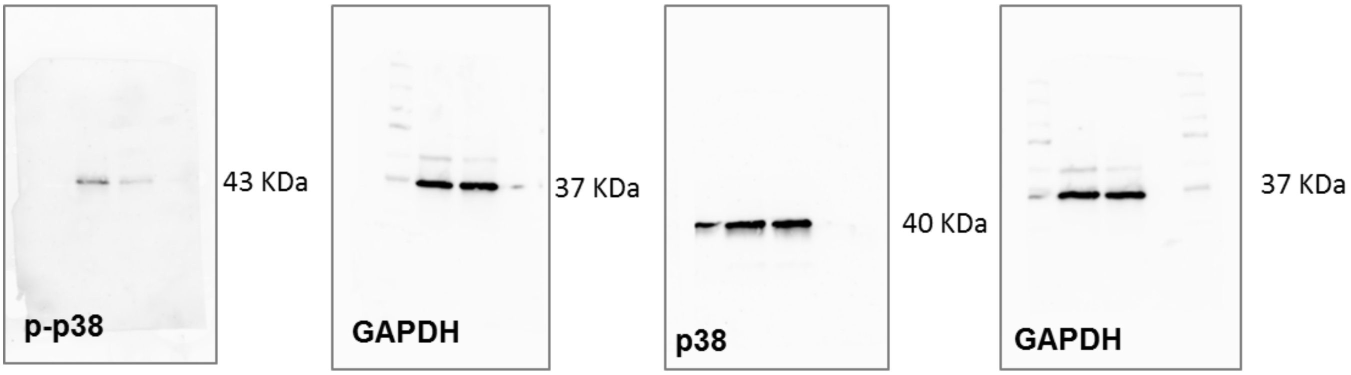

Fig. S5. Raw blots of western blots from Fig. 5E.

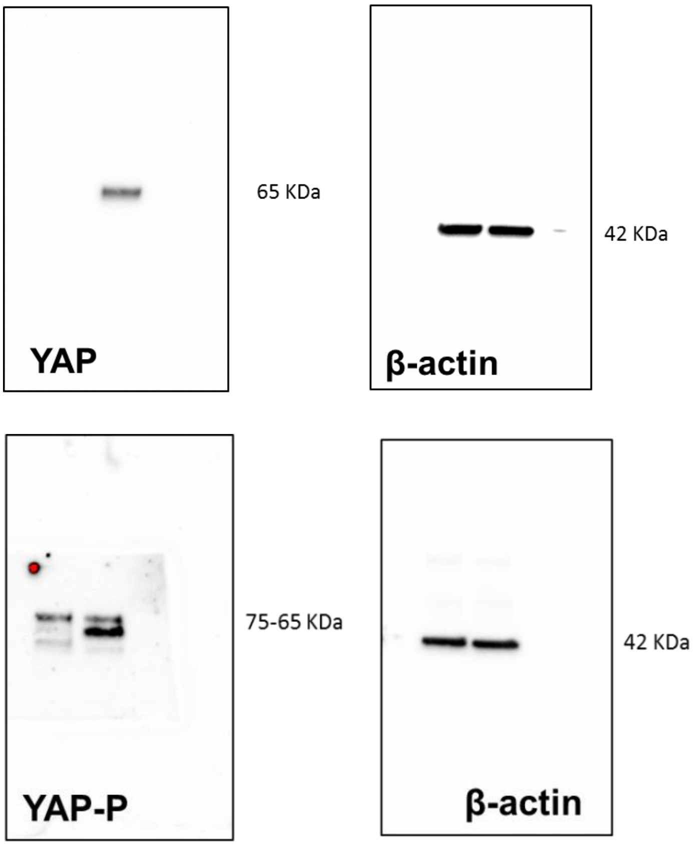

Fig. S6. Raw blots of western blots from Fig. 6D
